# Fat-soluble vitamins as biomarkers of nutritional status and their relation with complications in polytrauma patients

**DOI:** 10.1177/02601060241273640

**Published:** 2024-08-19

**Authors:** Esmee AH Verheul, Ebru Horzum, Suzan Dijkink, Pieta Krijnen, Jochem M Hoogendoorn, Sesmu M Arbous, Ron Peters, Inger B Schipper

**Affiliations:** 1 Department of Trauma Surgery, 4501 Leiden University Medical Centre, Leiden, The Netherlands; 2 Department of General Surgery, 2901 Haaglanden Medical Centre, The Hague, The Netherlands; 3 Acute Care Network West Netherlands, Leiden, The Netherlands; 4 Department of Intensive Care, 4501 Leiden University Medical Centre, Leiden, The Netherlands; 5 Department of Intensive Care, 2901 Haaglanden Medical Centre, The Hague, The Netherlands

**Keywords:** Nutrition, malnutrition, vitamins, trauma, biomarkers

## Abstract

**Background and Aims:**

This exploratory observational prospective study aimed to evaluate fat-soluble vitamin plasma levels during hospital admission and its relation with the development of malnutrition and complications in polytrauma patients, considering the protocolized multivitamin supplementation during intensive care unit (ICU) admission.

**Methods:**

In 49 well-nourished polytrauma (injury severity score ≥ 16) patients admitted to the ICU of two level-1 trauma centers, vitamin A, D, and E levels were assessed weekly during hospital stay. All patients received multivitamin supplementation during ICU stay. Linear mixed-effect models were used to assess a trend in vitamin levels over time during hospital stay. Mixed-effects logistic regression analysis was performed to relate vitamin concentrations with malnutrition, defined as a subjective global assessment score ≤5, and complications.

**Results:**

Vitamin A levels increased 0.17 µmol/L per week (95% confidence interval 0.12–0.22, p < 0.001), vitamin D levels increased 1.49 nmol/L per week (95% confidence interval 0.64–2.33, p < 0.01), vitamin E levels increased 1.17 µmol/L per week (95% confidence interval 0.61–1.73, p < 0.001) during hospital stay (29 ± 17 days). Vitamin levels were not related to malnutrition or complications during hospital stay.

**Conclusion:**

Vitamin A, D, and E levels increased due to supplementation during hospital admission. Plasma levels of vitamins A, D, and E do not seem to be useful as biomarkers for the nutritional status of polytrauma patients during hospital stay. No correlation with complications could be demonstrated.

## Introduction

Malnutrition is a serious problem during hospital admission, as it is known to be associated with adverse events, such as increased risk of mortality, infections, and increased hospital stay (Lochs et al., 2006; Ruiz et al., 2019). Research has shown that up to 50% of hospitalized patients were malnourished, depending on the patient population and definition used for diagnosis (Barker et al., 2011). Severely injured patients are even more susceptible for malnutrition, because of their hypermetabolic state after severe trauma, with an estimated prevalence range from 7% to 76% (Dijkink et al., 2020; Mogensen et al., 2015; Thomas et al., 2007). Nevertheless, objective assessment of the nutritional status of severely injured patients remains a major challenge (Kalaiselvan et al., 2017; Van Bokhorst-de van der Schueren et al., 2014; Yeh et al., 2018).

Potential new biomarkers for the assessment of nutritional status in severely injured patients are vitamins A, D, and E. These fat-soluble vitamins play a substantial role in a multitude of physiological processes (Reddy and Jialal 2021). For example, vitamin A aids in vision, vitamin D is important for bone mineralization, and vitamin E is known for its antioxidant properties (Reddy and Jialal 2021). A deficiency in these vitamins may have a significant impact on recovery after trauma and could increase the risk of several complications during hospital admission (Berger et al., 2022). Vitamin A deficiency can impair the humoral defence mechanism during the inflammatory phase in the wound-healing process, as vitamin A plays a key role in the differentiation, migration and development of T cells, modulates the balance between Th1 and Th2 immunity, and induces transcriptional and functional changes in natural killer cells leading to altered metabolism (Džopalić et al., 2021; Jeong et al., 2024; Ross 2012). This may reduce (re)epithelialization, collagen synthesis, and granulation tissue development in the proliferative and remodeling phases (Polcz and Barbul, 2019; Zhang et al., 2019). Vitamin D is specifically involved in bone metabolism and immune response modulation (Gorter et al., 2014; Muresan et al., 2022). A vitamin D deficiency may lead to decreased bone mineral density, fragile bones, and a higher risk of fractures (Han et al., 2022). Furthermore, vitamin D has protective effects on the innate immune system and inhibitory effects on adaptive immunity (Džopalić et al., 2021). Concerning in-hospital outcomes, vitamin D deficiency increases susceptibility for severe infections and mortality, and low vitamin D receptor levels were found to be associated with high mortality in critically ill patients (De Haan et al., 2014; Erdoğan and Fındıklı, 2021). Vitamin E is best known for its antioxidant qualities, by inhibiting the generation of reactive oxygen species during the fat oxidation and the propagation of free radical reactions (Reddy and Jialal 2021; Rizvi et al., 2014). Furthermore, it regulates gene expression and transcription of, for example, connective tissue growth factor. As a result, immunity against soft tissue infections is enhanced by vitamin E (Hobson 2016; Pierpaoli et al., 2011).

Although several studies concluded that fat-soluble vitamin deficiencies might be related to malnutrition in hospitalized patients, no studies regarding this topic in the polytrauma patient population were found (Verheul et al., 2024). The goal of this exploratory study was to evaluate the trend in plasma vitamin A, D, and E levels during hospital admission and its relation with the development of malnutrition and complications in polytrauma patients, considering the protocolized multivitamin supplementation during intensive care unit (ICU) admission.

## Material and methods

This study was conducted at two level-1 trauma centers as part of the Malnutrition in Polytrauma Patients (MaPP) study (Dijkink et al., 2019). The MaPP study is an observational cohort study, that prospectively included adult (≥18 years) patients with severe injuries (“polytrauma,” defined as severity injury score ≥16), who were admitted to the ICU. Included patients had to be primarily managed in one of the participating hospitals and needed to be admitted to the ICU for ≥48 hours . Additionally, only patients who were well-nourished at admission, based on the subjective global assessment (SGA), were included. Patients with penetrating injuries or burn wounds were excluded. The study was conducted according to the Declaration of Helsinki guidelines and approved by the local Institutional Review Boards (protocol number: NL64016.058.17). Informed consent was obtained from the patients or their legal representative at the day of ICU admission or as soon as possible after that day. Patient inclusion started in July 2018 and ended in April 2022. This study has been reported in line with the Strengthening the Reporting of Observational Studies in Epidemiology (STROBE) Statement (von Elm et al., 2007).

As described in the study protocol, the a priori sample size calculation showed that 195 patients were needed to show a difference in complication rate between the groups with and without malnutrition (Dijkink et al., 2019). Due to the slow inclusion rate during the COVID-19 pandemic, however, it was decided to prematurely end the inclusion at 100 patients. For this study, all patients admitted to two of the five participating centers and of whom blood samples were available for vitamin analysis were included.

**Figure 1.**
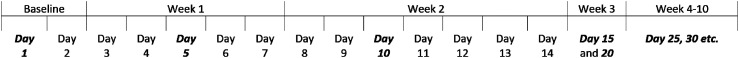
Combining the weekly vitamin concentration measurements with the Subjective Global Assessment. Bold and italic = Day of nutritional assessment using the Subjective Global Assessment.

A weekly plasma sample was drawn at the ICU or ward for the determination of plasma levels of vitamins A (retinol), D (25-hydroxy-vitamin D), and E (tocopherol). The samples were stored at the Clinical Chemistry and Laboratory Medicine (CCLM) department of the corresponding hospital at −80**°**C, transported on dry-ice if necessary, and analysed by the CCLM. The vitamin concentrations were matched with the corresponding SGA score assessed within 24 hours , and clustered into time periods (Figure 1). The first week was divided into the first 48 hours  (baseline period, the hyper acute early phase) and days 3 to 7 (week 1, subsequent period of metabolic instability and catabolism). After that, every week was a new time period (period of anabolism) (Singer et al., 2019).

All ICU patients received daily oral multivitamin supplementation (Supradyn, containing 800 µg vitamin A, 5 µg vitamin D and 12 mg vitamin E per tablet) for a minimum of 5 days upon admission. The multivitamin supplementation was continued as long as the patient received tube feeding. One patient received additional vitamin D supplementation other than the multivitamin supplementation. For this patient, the vitamin determinations after this supplementation were excluded from analyses.

Nutritional status, the primary outcome measure, was assessed using the SGA. The SGA is recommended as assessment tool for the nutritional status in the critically ill, since it is validated as a screening tool for predicting outcomes in the ICU and in the severely injured population (Fontes et al., 2014). Patients are classified as A: well-nourished (scores 6–7), B: mild/moderate malnutrition (scores 3–5), and C: severe malnutrition (scores 1–2). Following the general consensus, patient scores were divided into two categories: well-nourished (SGA category A, scores 6–7) and malnourished (SGA categories B and C, scores 1–5). The SGA was scored within 24 hours  after ICU admission to determine pre-existing malnutrition, and exclude the patients who were already malnourished upon admission. After that, the SGA was scored every 5 days at the ICU during ICU stay, on the day of discharge from the ICU, every week on the ward, and on hospital discharge day to determine in-hospital developed malnutrition. All SGA scores were determined by trained personnel, including a dietician, nurse, and a member of the research team.

Complications included systemic complications (sepsis, acute respiratory distress syndrome, systemic inflammatory response syndrome, and multiple-organ failure), surgery-related complications (such as wound infection), pneumonia, urinary tract infection, deep venous thrombosis, pulmonary embolism, fracture-related complications (such as compartment syndrome and fat embolism syndrome), and in-hospital mortality (Dijkink et al., 2019). The patients’ clinical files were checked for the presence of a complication on the day of vitamin analysis.

The data was analyzed using IBM SPSS Statistics and R version 4.2.2.

P-values <0.05 were regarded as statistically significant. The characteristics of the patients who developed malnutrition during hospital admission were compared with those of patients who remained well-nourished. Continuous variables were presented as mean with standard deviation (SD) and categorical variables were presented as frequency with percentage.

In the linear mixed-effects model analyses, the trends of the vitamin A, D, and E concentrations over time during hospital admission were analysed. Vitamin concentration was the dependent variable, time period (according to Figure 1) was the fixed effect, and patient was included as random effect.

Mixed-effects logistic regression analysis with repeated measures was performed to test the ability of each vitamin to determine malnutrition in each time period (Figure 1). For each vitamin, a baseline mixed-effects logistic regression model was fitted with malnutrition as binary outcome variable, time period as fixed effect, and patient as random effect. Then, a second mixed effects logistic regression model was fitted by adding vitamin levels and an interaction term of vitamin level with time period as fixed effects (full model). The interaction term was added to allow for changes in the association between vitamin levels and malnutrition over time. The likelihood ratio test was used to test the association between each of the vitamins and malnutrition over time.

Similar mixed-effects logistic regression analyses were performed to assess whether there was an association between each of the vitamins and complications over time.

## Results

Of the 100 patients included in the MaPP study, 51 patients were included in the two centers participating in this study. Two patients were malnourished at admission, and therefore data was collected from 49 patients who were well-nourished at admission. The mean age of the included patients was 50 years (range 18–85 years). Eight patients died during hospital admission (16%). Table 1 shows the baseline characteristics for the patients who remained well-nourished (SGA > 5; n = 12) and patients who became malnourished (SGA ≤ 5; n = 37) during their hospital stay. Forty patients (82%) received mechanical ventilation during ICU admission, which did not differ between the two groups. In addition, 43 patients (88%) received enteral feeding. This percentage seemed higher in the patients who developed malnutrition compared to the patients who remained well-nourished (92% vs 75%), but this difference was not statistically significant (p = 0.15). Patients who became malnourished during hospital stay had a significantly longer ICU length of stay (LOS) (12 ± 10 vs 5 ± 2 days, p < 0.001) and hospital LOS (32 ± 17 vs 17 ± 5 days, p < 0.001), and more ventilator days (9 ± 8 vs 2 ± 1, p < 0.001) than the patients who remained well-nourished during admission. In-hospital mortality in the patients who became malnourished seemed higher (33% vs 11%), but this difference was not statistically significant (p = 0.09).

**Table 1.** Patient characteristics according to their nutritional status during hospital admission.

Characteristic	Total (n = 49)	Developed malnutrition during admission (n = 37)	Remained well-nourished during admission (n = 12)	p-Value
Age in years, mean ± SD	50 ± 20	49 ± 20	53 ± 18	0.52
Female sex, n (%)	16 (33)	14 (38)	2 (17)	0.29
BMI in kg/m^2^, mean ± SD	26 ± 5	26 ± 5	27 ± 5	0.32
Obesity (BMI > 30 kg/m^2^), n (%)	9 (18)	6 (15)	3 (25)	0.67
Injury severity score ≥25, n (%)	40 (82)	31 (84)	9 (75)	0.67
Glasgow coma scale <8, n (%)	24 (49)	19 (51)	5 (42)	0.74
Mechanical ventilation, n (%)	40 (82)	31 (84)	9 (75)	0.67
Type of feeding, n (%)				0.15
* Only oral*	6 (12)	3 (8)	3 (25)	
* Enteral*	43 (88)	34 (92)	9 (75)	
* Parenteral*	0 (0)	0 (0)	0 (0)	
Alcohol abuse, n (%)	8 (16)	6 (16)	2 (17)	1.00
Malignancy, n (%)	3 (6)	2 (5)	1 (8)	1.00
In-hospital mortality, n (%)	8 (16)	4 (11)	4 (33)	0.09
ICU LOS in days *, mean ± SD	11 ± 9	12 ± 10	5 ± 2	<0.001
Ventilator days *, mean ± SD	7 ± 8	9 ± 8	2 ± 1	<0.001
Hospital LOS in days **, mean ± SD	29 ± 17	32 ± 17	17 ± 5	<0.001

*Patients who died during ICU admission were excluded (n = 6).

**Patients who died during hospital admission (n = 8) or were transferred to another hospital (n = 2) were excluded.

BMI: body mass index; ICU: intensive care unit; LOS: length of stay; n: number; SD: standard deviation.

Vitamin A levels increased with 0.17 µmol/L per week (95% confidence interval (CI) 0.12–0.22, p < 0.001), vitamin D levels increased 1.49 nmol/L per week (95% CI 0.64–2.33, p < 0.01), and vitamin E levels increased 1.17 µmol/L per week (95% CI 0.61–1.73, p < 0.001) during hospital stay.

**Figure 2.**
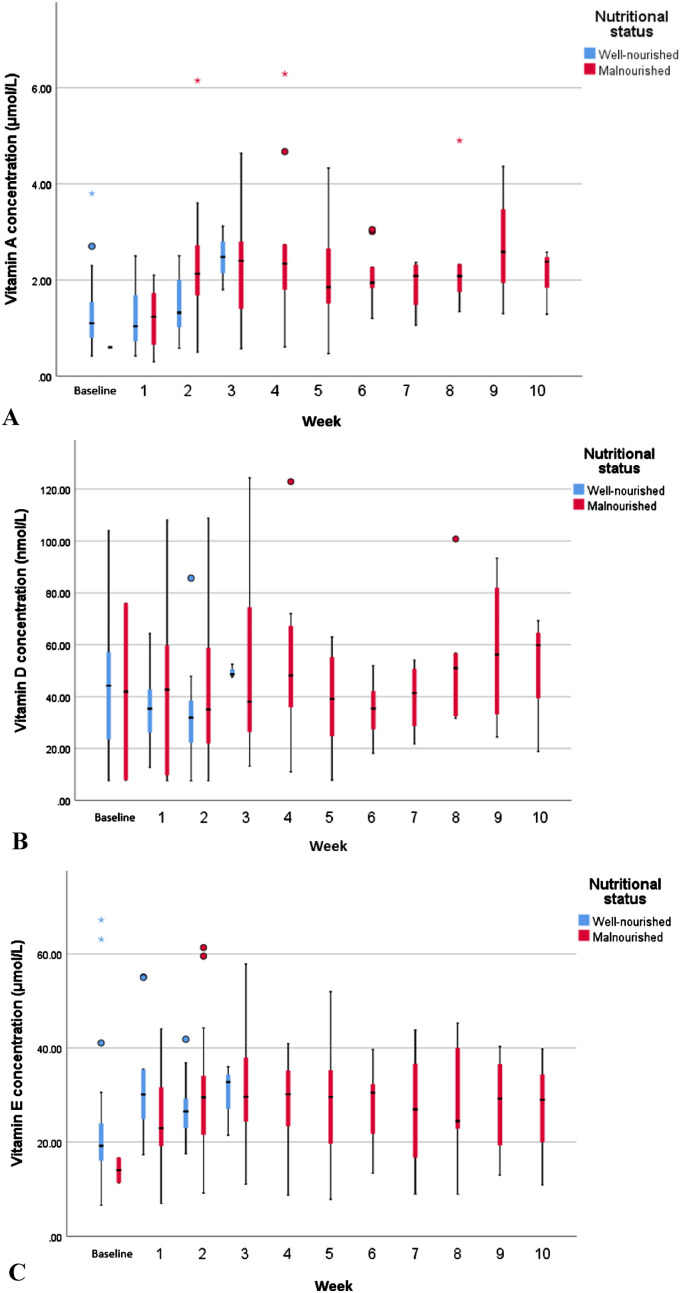
Boxplots of vitamin A (a), vitamin D (b), and vitamin E (c) levels by time and nutritional status during hospital stay. From week 7 onwards, each period included <6 patients. The boxes represent the median vitamin concentrations with the first and third quartile. The whiskers show the minimum and maximum vitamin levels.

The boxplots in Figure 2 show the vitamin concentration distributions for the well-nourished and malnourished patients in each time period during hospital admission. After 3 weeks of admission, all the patients who were still admitted (n = 25) had become malnourished and remained malnourished until discharge. Vitamin A, D, and E levels did not predict malnutrition (Table 2). For all vitamins, the likelihood ratio test between the base model (time only) and the full model (with time and malnutrition) was not statistically significant (p = 0.45, 0.62, and 0.34, respectively).

**Table 2.** Mixed-effects logistic regression analysis predicting malnutrition during hospital admission based on vitamin concentration and time.

Vitamin		Odds ratio (95% CI)	p-Value	LR test, p-value
Vitamin A	Time period	**81 (1.28–5087)**	**0**.**04**	0.45
	Main effect VitA	2.64 (0.22–32)	0.45	
	Interaction effect	0.93 (0.24–3.56)	0.91	
Vitamin D	Time period	**64 (1.23–3276)**	**0**.**04**	0.62
	Main effect VitD	1.01 (0.92–1.10)	0.86	
	Interaction effect	1.01 (0.96–1.07)	0.61	
Vitamin E	Time period	**366 (1.54–86,759)**	**0**.**03**	0.34
	Main effect VitE	1.17 (0.91–1.51)	0.22	
	Interaction effect	0.95 (0.85–1.07)	0.43	

LR test comparing base model (including only Time period) and full model. Results with a p > 0.05 is bold.

CI: confidence interval; LR: likelihood ratio.

No association was found between vitamin A, D, and E levels and complications over time (Table 3). For all vitamins, the likelihood ratio test between the base model (time only) and the full model (with time and complications) was not statistically significant (p = 0.60, 0.76, and 0.78, respectively).

**Table 3.** Mixed-effects logistic regression analysis predicting complications during hospital admission based on vitamin concentration and time.

Vitamin		p-Value	Odds ratio (95% CI)	p-Value LR test
Vitamin A	Time period	0.53	1.12 (0.80–1.56)	0.60
	Main effect VitA	0.79	0.92 (0.50–1.70)	
	Interaction effect	0.70	0.97 (0.82–1.14)	
Vitamin D	Time period	0.42	1.14 (0.83–1.56)	0.76
	Main effect VitD	0.68	1.00 (0.98–1.03)	
	Interaction effect	0.48	1.00 (0.99–1.00)	
Vitamin E	Time period	0.44	1.14 (0.82–1.60)	0.78
	Main effect VitE	0.56	1.01 (0.97–1.06)	
	Interaction effect	0.49	1.00 (0.98–1.01)	

LR test comparing base model (including only time period) and full model.

CI: confidence interval; LR: likelihood ratio.

## Discussion

In this exploratory study, an increase in vitamin A, D, and E levels was observed during hospital admission, but no association was found between vitamin A, D, and E levels and nutritional status or complications in polytrauma patients.

This is the first study to evaluate the trend in fat-soluble vitamin levels during hospital admission in polytrauma patients. Polytrauma patients suffer from an acute phase response after severe trauma, with a hypermetabolic state (Dijkink et al., 2020). Previous research in hospitalized patients has shown that a systemic inflammatory response, defined by increased C-reactive protein (CRP) levels, influences plasma vitamin levels (Duncan et al., 2012). Inflammation causes an increase of capillary permeability, leading to a redistribution of retinol binding protein and vitamin D binding protein into the interstitial fluid. Since retinol binding protein and vitamin D binding protein are also considered to be negative acute phase proteins, inflammation causes a decrease in vitamin A (retinol) and vitamin D levels, respectively (Berger et al., 2022; Duncan et al., 2012; Hernández-Álvarez et al., 2019; Waldron et al., 2013). Vitamin E levels are less affected by inflammation, because of the large size of the lipoprotein in which vitamin E is incorporated, which limits its transfer across capillaries into the extravascular space (Berger et al., 2022; Duncan et al., 2012). In a small study including patients undergoing limb surgery, a decrease in serum vitamin A, D, and E levels was demonstrated around 24 to 48 hours  after surgery, alongside with a peak in CRP levels. Subsequently, the vitamin levels increased and CRP levels decreased substantially in the days that followed (Louw et al., 1992). In our study, an increase in vitamin A, D, and E levels is demonstrated during hospital admission. Our study population received multivitamin supplementation according to a standardized hospital protocol for a minimum of 5 days during ICU admission, which explains the significant increase in vitamin levels. It can be hypothesized that all polytrauma patients, without vitamin supplementation, might have become deficient for all three vitamins. This suggest that the standardized supplementation protocols used, sufficiently elevated the plasma vitamin levels in ICU admitted polytrauma patients.

Concerning the relation between plasma vitamin levels and malnutrition, several studies have been performed involving other types of hospitalized patients (Verheul et al., 2024). Terlikowska et al. found no relation between vitamin E levels and moderate or severe malnourishment in women who were surgically treated for patients with ovarian cancer, but did find that the malnourished patients had significantly lower levels of vitamin A than the well-nourished patients (Terlikowska et al., 2021). A study by Cunha et al. (2001) found no association between vitamin A and E levels and malnutrition in hospitalized patients aged older than 65 years. In addition, several other studies found no relation between vitamin D levels and malnutrition in hospitalized patients (Christopher et al., 2013; Førli et al., 2009; Holden et al., 2010; Olvera-Soto et al., 2018; Ramel et al., 2009; Vischer et al., 2012; Vosoughi et al., 2016). On the other hand, previous studies on patients with fractures found that the risk of malnutrition was correlated with decreased vitamin D levels (Li et al., 2018; Tsagari et al., 2012). In our study, no relation was found between plasma vitamin A, D, and E levels and in-hospital developed malnutrition. However, comparing our findings to those of the referenced studies poses challenges due to the difference in type of malnutrition. Our study population developed malnutrition caused by “acute injury states with marked inflammatory response,” while the cited studies included patients enduring “malnutrition as a result of chronic disease,” such as malignancies (Jensen et al., 2010). As stated earlier, our patients suffer from inflammation, which might also influence the results. In addition, the supplementation obscures the potential vitamin deficiencies, that might correlate with malnutrition.

Several nutritional assessment tools are available to diagnose malnutrition, although no “gold standard” has been established. We used the SGA score to diagnose malnutrition in the polytrauma patients in our study, as it is validated in the critically ill population. In 2019, after the start of our study, a consensus report was published by the Global Leadership Initiative on Malnutrition (GLIM) (Cederholm et al., 2019), pointing out that the nutritional status of hospitalized patients should be assessed according to the GLIM diagnostic criteria for malnutrition. These criteria include: (1) nonvolitional weight loss, (2) low body mass index (BMI), (3) reduced food intake or assimilation, (4) disease burden/inflammation, and (5) reduced muscle mass. The SGA score entails weight (change) (point 1), dietary intake and gastrointestinal symptoms (point 3), disease state (point 4), and physical examination (point 5) (Cederholm et al., 2017; Detsky et al., 1987). The SGA criteria match those of the GLIM to a large degree, since only low BMI is not included in the SGA. However, according to the GLIM consensus statement, the BMI is seldom used as a clinical malnutrition marker in North America, since the American population is often overweight or obese (Cederholm et al., 2017). The SGA score thus largely fulfills the GLIM diagnostic criteria for malnutrition. In line with the literature, we found that all polytrauma patients who were admitted for at least three weeks, became malnourished (Amarya et al., 2015; Fávaro-Moreira et al., 2016; Volkert et al., 2019).

The ESPEN Micronutrient guideline describes that patients with vitamin A deficiency are more susceptible for respiratory tract infections and that low vitamin E levels could make a patient more susceptible for infections (Berger et al., 2022). Several studies suggest that adequate vitamin A, D, and E levels play an important protective role in septic patients due to its role in the regulation of inflammatory responses against infection (Berger et al., 2022; Herr et al., 2011; Izadpanah and Khalili 2013). Moreover, the study of Takeuti et al. recommended vitamin D supplementation against sepsis prevention and sepsis treatment (Takeuti et al., 2018). The inflammatory response triggered by sepsis can disrupt the normal metabolism of vitamins A, D, and E (Wald et al., 2022). This is in line with other studies, that show that vitamins A, D, and, E are commonly lower in septic patients (Chae et al., 2023; Corcoran et al., 2009; Delrue et al., 2023; Li et al., 2019; Zhang et al., 2019). We did not find a relation between vitamin levels and complications. One of the reasons for not finding a relation might be the heterogeneity of this patient population. The age of the included patients ranged from 18 to 85 years old, including previously healthy patients and patients with several comorbidities. For example, kidney failure causes an increase in vitamin A levels as it impairs the degradation of retinol binding protein. Vitamin D levels on the other hand, tend to decrease with progressive loss of kidney function (Juszczak et al., 2023). In addition, vitamin A and D levels tend to influence each other, as vitamin A and D act through a similar type of receptor. In case of hypervitaminosis A, vitamin D supplementation is less effective (Džopalić et al., 2021). Since multiorgan failure in polytrauma patients may also induce progressive kidney failure, this should be taken into account when supplementing vitamins in polytrauma patients.

Due to daily vitamin supplementation during ICU stay, vitamin A, D, and E levels increased during hospital admission. The vitamin levels were not related to malnutrition or complications during hospital stay nor could a correlation with complications be demonstrated. Thus, these fat-soluble vitamins do not seem to be useful as biomarkers for the nutritional status or complications of polytrauma patients in clinical practice. More research could help establish the detailed relation between fat-soluble vitamins and nutritional status and whether additional supplementation of these vitamins plays a role in reducing the risk of developing hospital complications.

This study has several limitations. A gold standard to determine malnutrition does not exist worldwide. In this study, the SGA was used to determine the nutritional status of the polytrauma patients, which is a reliable assessment tool and recommended by the European Society for Clinical Nutrition and Metabolism (ESPEN) (Lochs et al., 2006; Norman et al., 2005). However, a limitation of the SGA is its accuracy in relation to the experiences of the person conducting the SGA, due to the minimal difference between an SGA score of 5 (malnourished) and an SGA score of 6 (well-nourished) (Xu and Vincent, 2020). On account of this limitation, the measured SGA was verified by another person in order to minimize the risk of bias. Additionally, with only 49 participants the statistical power of this study is low, potentially causing clinically relevant differences between patient groups to remain statistically not significant. As stated earlier, the heterogeneity of the study population is a limitation. Intubation is known to result in significant tracheal inflammation and sepsis can disrupt the normal metabolism of fat-soluble vitamins (Puyo and Dahms 2012; Wald et al., 2022). We were not able to correct for these confounders because of the low sample size. Future studies with larger patient cohorts could take several confounders into account, such as comorbidities, mechanical ventilation, type of feeding, and CRP level. The ESPEN micronutrient guideline states that vitamin A, D, and E levels become less interpretable with high CRP values (Berger et al., 2022).

## Conclusion

This exploratory prospective study showed that plasma levels of vitamins A, D, and E do not seem to be useful as biomarkers for the nutritional status of polytrauma patients during hospital stay.

## Supplemental Material

sj-docx-1-nah-10.1177_02601060241273640 - Supplemental material for Fat-soluble vitamins as biomarkers of nutritional status and their relation with complications in polytrauma patients Supplemental material, sj-docx-1-nah-10.1177_02601060241273640 for Fat-soluble vitamins as biomarkers of nutritional status and their relation with complications in polytrauma patients by Esmee AH Verheul, Ebru Horzum, Suzan Dijkink, Pieta Krijnen, Jochem M Hoogendoorn, Sesmu M Arbous, Ron Peters and Inger B Schipper in Nutrition and Health
